# Self-assembled levitating clusters of water droplets: pattern-formation and stability

**DOI:** 10.1038/s41598-017-02166-5

**Published:** 2017-05-15

**Authors:** Alexander A. Fedorets, Mark Frenkel, Evgeny Shulzinger, Leonid A. Dombrovsky, Edward Bormashenko, Michael Nosonovsky

**Affiliations:** 1 Tyumen State University, 6 Volodarsky St., Tyumen, 625003 Russia; 2 Department of Physics, Natural Sciences Faculty, Ariel University, Ariel, 40700 Israel; 3 Chemical Engineering, Biotechnology and Materials Department, Engineering Faculty, Ariel University, Ariel, 40700 Israel; 4 Joint Institute for High Temperatures, 17A Krasnokazarmennaya St., Moscow, 111116 Russia; 5 Mechanical Engineering, University of Wisconsin-Milwaukee, 3200 N Cramer St., Milwaukee, WI 53211 USA

## Abstract

Water forms ordered hexagonally symmetric structures (snow crystals) in its solid state, however not as liquid. Typically, mists and clouds are composed of randomly moving small droplets lacking any ordered structure. Self-organized hexagonally patterned microdroplet clusters over locally heated water surfaces have been recently observed. However, many aspects of the phenomenon are far from being well understood including what determines droplets size, arrangement, and the distance between them. Here we show that the Voronoi entropy of the cluster tends to decrease indicating to their self-organization, while coupling of thermal effects and mechanical forces controls the stability of the clusters. We explain the balance of the long-range attraction and repulsion forces which stabilizes the cluster patterns and established the range of parameters, for which the clusters are stable. The cluster is a dissipative structure similar to self-organized Rayleigh–Bénard convective cells. Microdroplet formation plays a role in a variety effects from mist and clouds to aerosols. We anticipate that the discovery of the droplet cluster phenomenon and its explanation will provide new insights on the fundamental physical and chemical processes such as microdroplet role in reaction catalysis in nature as well as new tools for aerosol analysis and microfluidic applications.

## Introduction

Self-organized hexagonally patterned microdroplet clusters over locally heated water surfaces have been recently observed^1–4^. The clusters are dissipative structures similar to self-organized Rayleigh–Bénard convective cells^5^. However, many aspects of the phenomenon are far from being well understood including what determines the size of the droplets in the cluster, the distance between them, and the regular arrangement of the droplets.

The microdroplets of water play a significant role in a variety of processes from the water cycle and climate formation to microfluidics and possible catalytic effects in biochemical reactions including those of abiogenesis. Usually, the microdroplets are difficult to trace and they form random conglomerations such as clouds. However, stable clusters of levitating micro-droplets over a thin layer of heated water have been observed and studied in a remarkable series of recent experiments. These experiments showed that droplets self-assemble into a hexagonally patterned cluster levitating over the hottest part of the surface. Besides water, the phenomenon was also observed in different liquids including Glycerol, Benzyl alcohol, and Ethylene glycol^1, 3^.

## Results

**Figure 1:**
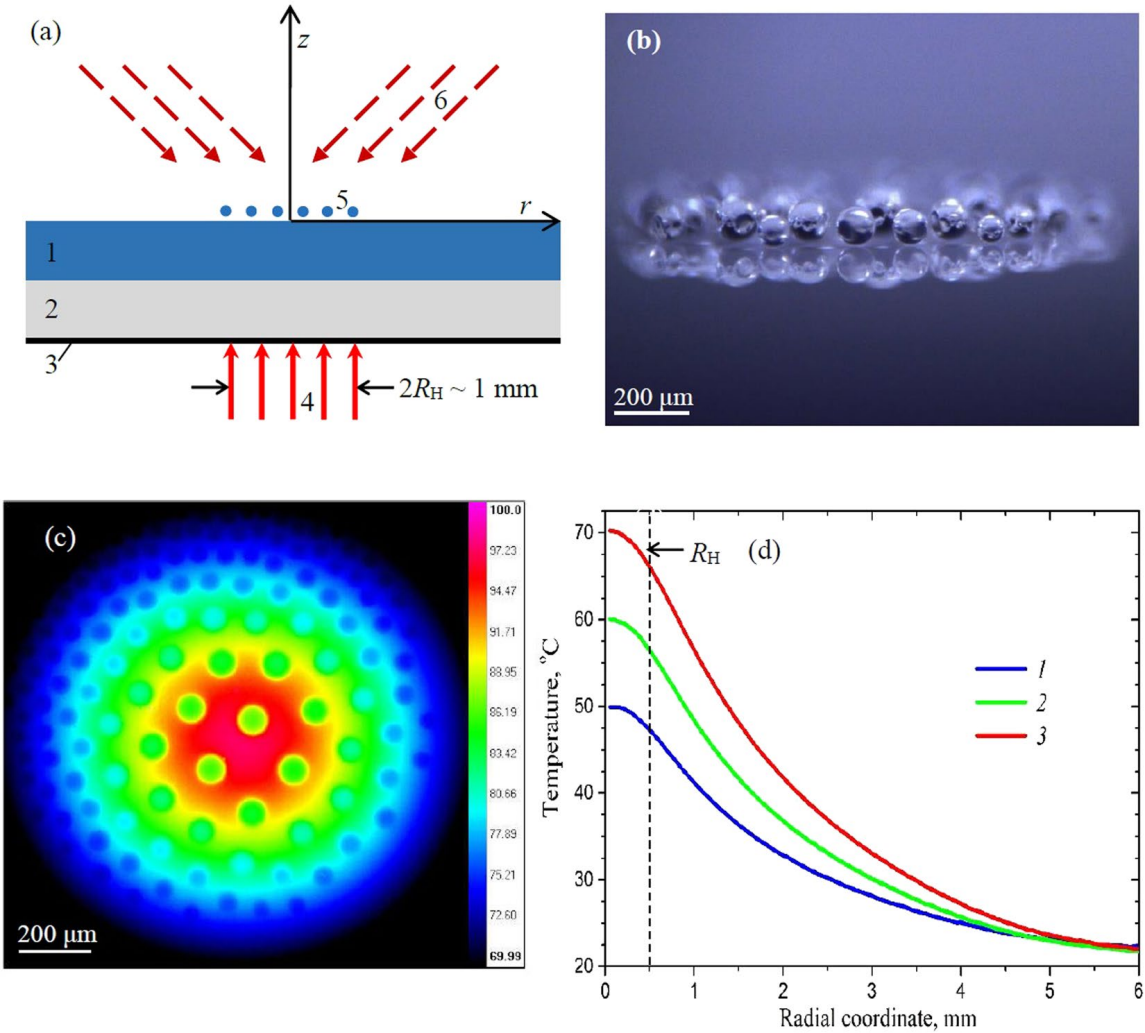
(**a**) The schematic of experimental setup: the water layer (1) on glass-ceramic substrate (2) with light-absorbing coating (3) is heated by laser beam (4) and generates droplet cluster (5) stabilized by infrared irradiation (6). (**b**) Side view of the cluster. (**c**) Top view, the infrared image and (**d**) radial temperature profiles of water surface under the cluster at the laser power of 208.6 mW (1), 297.1 mW (2), and 411.4 mW (3).

An experimental setup includes a cuvette with a submillimeter-thick layer of water (sometimes with an addition of surfactants to suppress bubbling or waving at the surface) heated locally from the bottom to reach the water surface temperature of 50–70 °C, either by laser irradiation and/or by a heating element at a spot of several millimeters in diameter (Fig. 1a). A monolayer of condensed micro-droplets emerges above the water layer (Fig. 1b). This is because local heating creates heat flux at the water surface (Fig. 1c,d) and the resulting upward vapor-air flow^4^. The cluster can also be stabilized by external infrared irradiation which significantly increases their time of existence^5^.

For a given temperature, the droplet radius *R* tends to increase with time^5^, often consistently with the *t* ^1/2^ law. The typical radius of levitating water droplets is dozens of microns, and they levitate at the height comparable with the droplet diameter. The radii of droplets are much smaller than the capillary length, which is 2.71 mm for water^6, 7^, thus droplets keep their spherical shape. The droplets do not coalescent with each other and with the water layer while they form a relatively stable cluster involving dozens or hundreds of droplets arranged in a distinct hexagonal (honeycomb) pattern. When new droplets enter the cluster from outside, they occupy a certain place in the structure maintaining the hexagonal arrangement. In certain cases, pairs (or tandems) of attached droplets enter the cluster zone and then detach from each other. The droplet size is controlled by modifying the maximum temperature and the size of the heated zone. A larger zone results in smaller droplets with a larger number of droplet and smaller distances between them. Increasing temperature has the opposite effect^1–5^.

While growing, the droplets approach the water surface. When the height of levitation becomes sufficiently small, the cluster can spontaneously collapse as a chain reaction: if one droplet touches the water layer and coalesces, a capillary wave is created which destroys other droplets as well within a millisecond^8^. In the case of an external infrared irradiation, the clusters exist for observation times longer than 10 minutes^5^.

The radii of the droplets in a central part of stable cluster are more or less equal to each other, with somewhat smaller droplets at the edges of the cluster where both the steam volume fraction and the gas temperature is reduced comparing to that at the center of the cluster. The distance, *L*, between the droplets is in the range between a fraction of the droplet diameter to several diameters and depends on many factors. When other conditions are identical, *L* is proportional to the difference between the surface temperatures of water layer at the hot center and cold periphery of the heating area. With growing diameter of the droplets the distance between their centers remains almost constant, as well as droplets position relative to each other, so it is possible to trace each droplet, while the levitating cluster as a whole can slide on top of the water surface, apparently following the fluctuations of the heat flux and local vapor flow rate. The main mechanism determining the droplet size is the dynamic balance between the evaporation and condensation^3, 5, 9^. The larger droplets are electrically neutral. It has been reported that a small charge of less than 10^−16^ C could accumulate at the initial stage of droplet formation^10^, although the electric forces are insufficient to affect them^11^. Therefore, the repulsion forces are most likely of the hydrodynamic nature. On the other hand, the exact nature of interactions between the droplets which results in the hexagonal shape of the cluster remains obscure.

**Figure 2:**
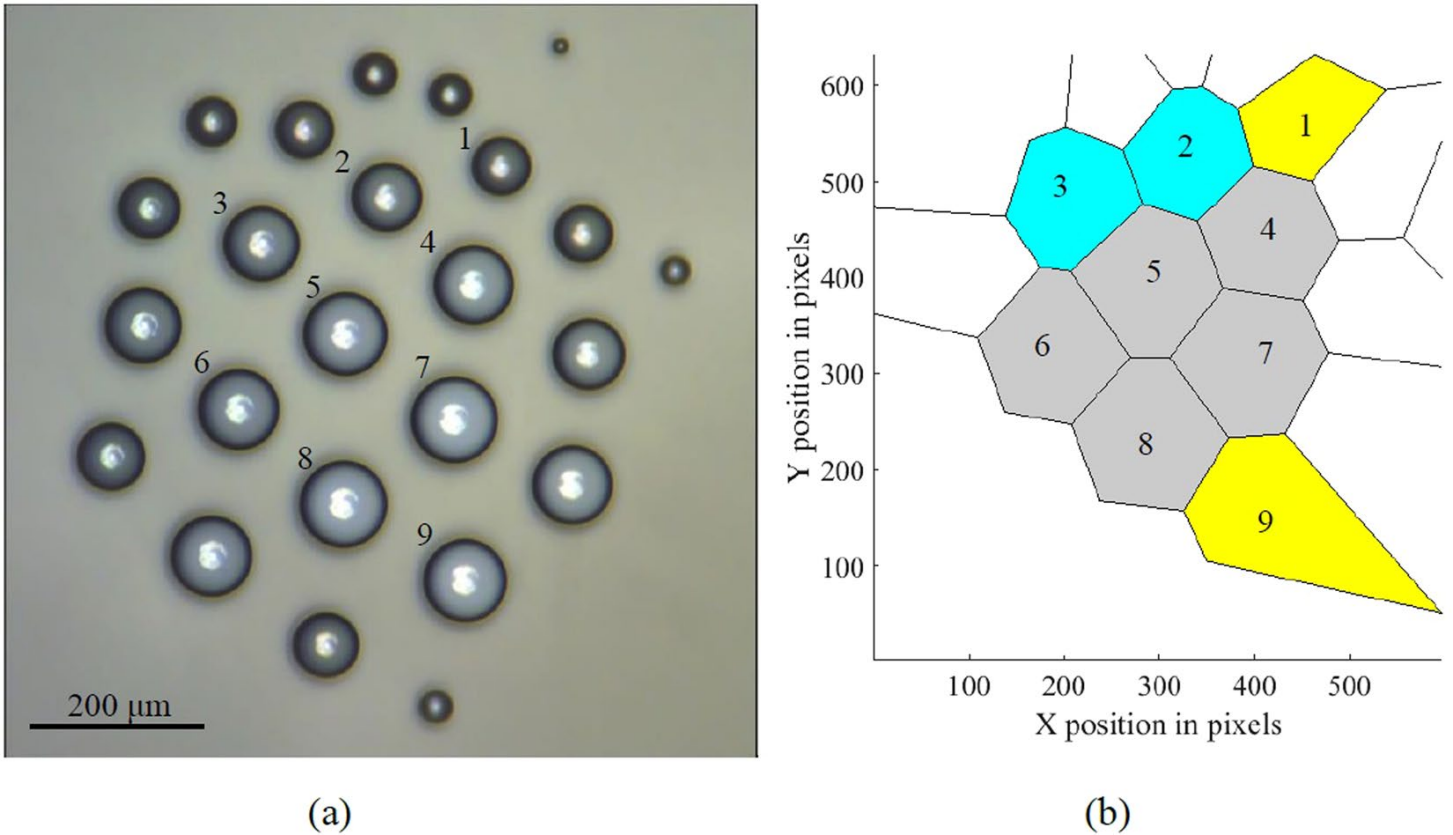
Self-organization of a droplet cluster. (**a**) The image of the cluster and (**b**) the Voronoi tessellation of the cluster. Yellow (1,9), gray (4–8), and blue (3,2) polygons have five, six, and seven neighbors, correspondingly.

Voronoi entropy can serve as a quantitative test of self-organizing behavior. A Voronoi diagram is a partition of a plane into cells nearest to specified points in the plane^12, 13^. Using the video images of the cluster (Fig. 2a), the Voronoi diagram was created (Fig. 2b), and a number of polygon cells with *m* neighbors, *N*_*m*_, was calculated. The Voronoi entropy is then defined as

1 $$S=-\sum _{m}{P}_{m}\,\mathrm{ln}({P}_{m})$$

where *P*_*m*_ = *N*_*m*_ */N* is the probability of a polygon and $N=\sum _{m}{N}_{m}$ is the total number of cells.

The time-dependent Voronoi entropy of cluster was calculated using the software developed in Department of Physics and Astronomy University of California, Irvine.

In general, we have three types of mechanical motion, which can be assumed decoupled: the levitation of the droplets in *z*-direction governed by the balance of the pressure gradient and the gravity; migration of the droplets towards the center of the hot spot in *r*-direction due to the coupling of the temperature and pressure gradients; and repulsion forces between the droplets. Together, these three effects result into the observed packing of levitating droplets near the center of the hot spot.

**Figure 3:**
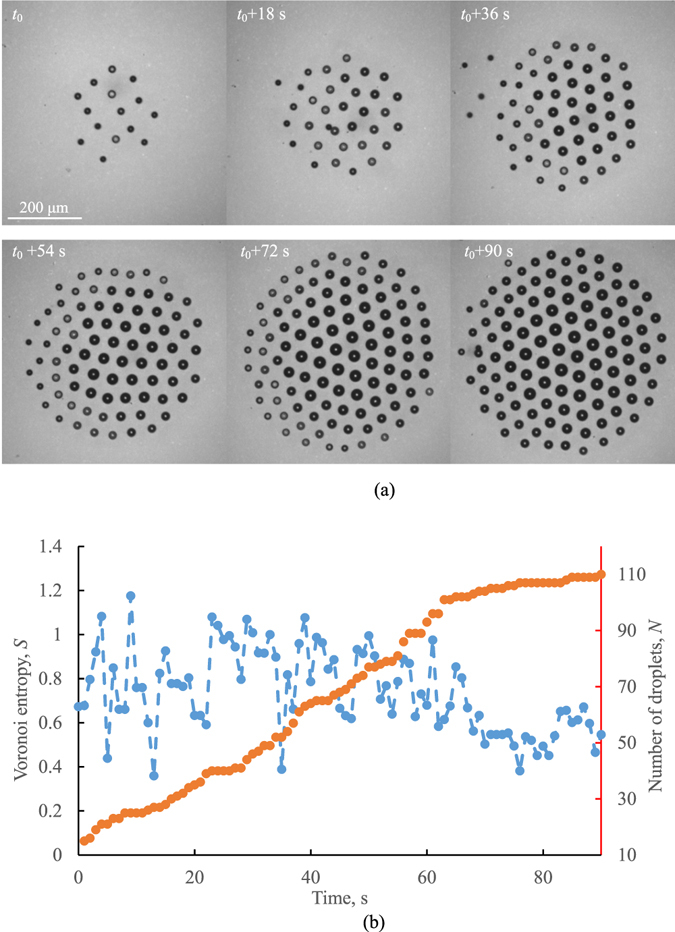
(**a**) Self-assembly of a droplet cluster over a heated water and (**b**) the Voronoi entropy, *S* (blue), correlated with the number of droplets, *N* (red).

A typical cluster at different time is shown in Fig. 3a. The results shown in Fig. 3(b) indicate a decrease in the Voronoi entropy with increasing time and the number of droplets. Newly arriving droplets disturb the hexagonal structure, and the size of the droplets affects the Voronoi entropy. When these new droplets grow, they affect the structure and result in the entropy growth. There are two opiate tendencies in the dependency of the entropy upon time. The Voronoi entropy tends to grow immediately after a new droplet joins the cluster; however, after that, due to the ordering of the cluster arrangement, the entropy tends to decrease. Note that the Voronoi entropy is an intensive property (unlike the thermodynamic entropy, which is an extensive property), and it is independent of the number of droplets and reflects only their ordering. Most test also showed a correlation between *S* and the fraction of hexagonal clusters, *P*_*6*_. This is because the hexagonal arrangement provides the densest 2D packing.

## Discussion

The phenomenon of droplet levitation is not uncommon; however, in most cases the levitation is caused by non-linear viscous properties of the liquid or gas layer, vibration, or by acoustic levitation^14^. Mathematically, the cluster can be described by coupled 3D differential equations of continuity, motion, and energy balance for both water in the droplets and for the gas (steam-air mixture around the droplets), and the diffusion equation for the gas mixture. In the case of droplet clusters stabilized by the external infrared irradiation, the radiative transfer equation should be included as well (the electromagnetic wave effects are expected to be negligible because the droplet size is much greater than the radiation wavelength). We will perform a simplified analysis first studying behavior of a droplet as an uncharged point mass in an axisymmetric flow field and then the interaction between two such point droplets.

A levitating droplet is subject of both thermal processes and mechanical forces. The steam/air flow above the heated water surface is characterized by an approximately axisymmetric steady-state distributions of the temperature *T*(*z,r*), pressure *p*(*z,r*), and steam concentration or relative humidity, *R*_*h*_(*z,r*). These distributions result in the heat flux, flow field and humidity patterns.

For small Reynolds numbers (in the Stokes flow regime), the drag force on a single particle of the mass $m={\rho }_{w}4\pi {R}^{3}/3$ with the radius *R* at the gas flow velocity *U* is given by 6*πμUR*, where μ is the dynamic viscosity, so the resulting force acting on the droplet is

2 $$\overrightarrow{F}=6\pi \mu \overrightarrow{U}R-{\rho }_{w}(\frac{4\pi }{3}){R}^{3}\overrightarrow{g}$$

It is assumed here that there is a conventional undisturbed/incident flow of gas in the direction opposite to the gravitational force. The equilibrium condition is then given by $R=\sqrt{\frac{9\mu U}{2{\rho }_{w}g}}$. For the kinematic viscosity of ν = 2 × 10^−5^ m^2^ s^−1^ and density is ρ = 1.01 kg m^−3^ (air at 75 °C), the dynamic viscosity is equal to μ = νρ = 2 × 10^−5^ kg m^−1^ s^−1^. Having substituted the values of *ρ*_*w*_ = 10^3^ kg m^−3^ and *U* = 0.1 m s^−1^, we obtain a typical droplet radius of *R* = 17 μm.

Coupling of a non-uniform temperature field and diffusion, referred to as the thermal diffusion or the Soret effect, is a classic example of coupled non-equilibrium processes observed on the sub-millimeter length scale^15^. The temperature gradient, ∇*T*, results also in a concentration gradient, ∇*c*, of a gaseous component of a quasi-steady multiphase system, $D\nabla c+{D}_{T}c(1-c)\nabla T=0$, where *D* is the diffusion coefficient and *D*_*T*_ is the thermal diffusion coefficient. Similarly, one can assume a linear dependency between the pressure and temperature gradients, *U*~∇P~∇T.

The droplet changes its volume due to the competitive condensation and evaporation. The rates of these processes depend on the relative humidity, *R*_*h*_, of ambient gas and on the current radius of the droplet. In accordance with the Kelvin equation, the equilibrium radius, *R*_*e*_, depends on the relative humidity according to formula $\mathrm{ln}\,{R}_{h}=2\gamma {V}_{m}/({R}_{e}{R}_{0}T)$, where γ is the surface tension, *V*_*m*_ is the molar volume (γ = 63.6 Jm^−2^, *V*_*m*_ = 0.0286 m^3^ at *T* = 75 °C), and *R*_0_ = 8.314 J mol^−1^ K^−1^ is the universal gas constant. An estimate of the droplet growth kinetics can be provided assuming that the condensation speed per unit area is inversely proportional to the difference of the droplet radius and the equilibrium radius *R* − *R*_*e*_. The volume change due to the condensation per unit time 4/3*πR* ^2^ *dR*/*dt* is also proportional to the droplet area 4*πR* ^2^/3:

3 $$\frac{dR}{dt} \sim 2\gamma {V}_{m}/[(R-{R}_{e}){R}_{0}T]$$

which yields the square root low *R*~*t* ^1/2^ providing a good fit with the experimentally observed trend^4, 5^.

Furthermore, the mechanical stability is governed by the reaction of the system to the change of droplets position. If, due to a random fluctuation, the droplet elevated from its original position *z* to *z* + δ*z*, this results in the change of the force acting upon the droplet based on Eq. 2 yielding the stability criterion

4 $$(\frac{\partial U}{\partial z}R+U\frac{\partial R}{\partial z}\,)-4{\rho }_{w}g{R}^{2}/(6\mu ) > 0\,$$

**Figure 4:**
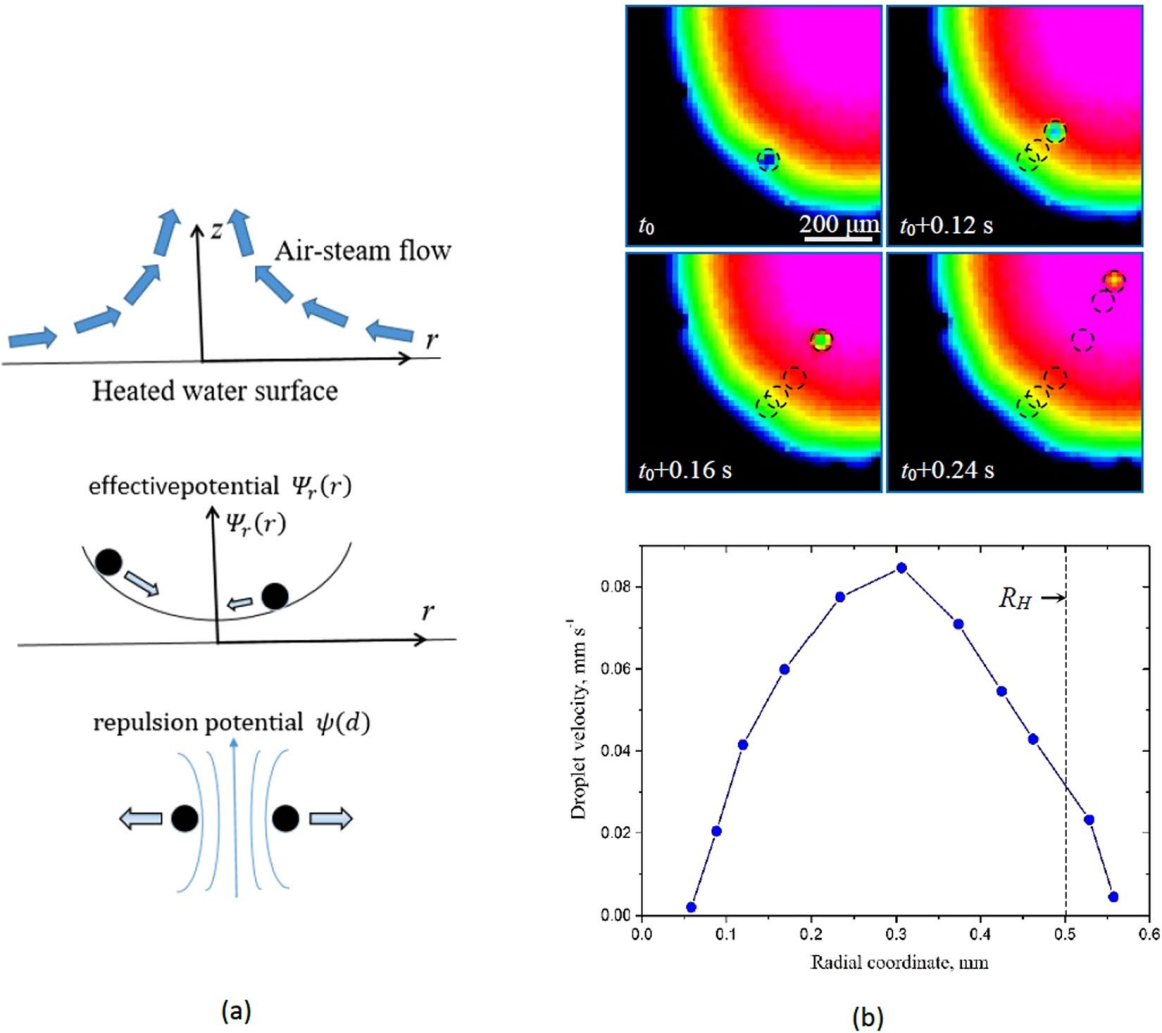
(**a**) The air-steam flow over the heated water surface creates pressure gradient causing droplets to levitate. The *r*-component of the drag force can be approximated with an effective potential and is minimal at the center. The repulsion force between the droplets can be presented as a repulsion potential. Together, dragging droplets to the center combined with the repulsion results in the observed packing of the droplets minimizing the total potential Ψ_*total*_ and thus leading to the self-assembly as evidenced by Voronoi entropy decrease. (**b**) Experimental observation of the velocity dependence on the distance from the center for a single droplet.

While the vertical position of the droplet is controlled by Eqs 2 and 4, the horizontal position is mostly driven by the radial component of temperature gradient (Fig. 4a). The radial component of the pressure gradient is a result of a more intensive evaporation at the hot center of the spot, higher speed of the air/vapor flow and decreased pressure. The radial force can be estimated as the Stokes force *F*_*r*_ = |grad *P*_*r*_| *V*_*d*_ = 12*πη*_*g*_ *dV*_*d*_, where *V*_*d*_ = 4*πR* ^3^/3 is the droplet volume, *d* = 2 *R*, and *η*_*g*_ = 18.5·10^−6^ Pa·s is the viscosity of the humid air. At the flow velocity *U* = 0.3 ms^−1^, the local pressure drop is Δ*Р* = *ρU* ^2^/2 ≈ 50 Pa, while the radial pressure gradient calculated from single droplet velocity observations is less than 16 Pa/mm. This corresponds to the drag force of 2 nN (an order of magnitude smaller then droplet’s weight), which is equilibrated by a repulsion force.

The droplet migrates towards the center of the heated spot (the hottest point) with typical velocities of 10–100 μms^−1^ (Fig. 4b). An effective 2D potential can be introduced with its minimum at the center of the hot spot by integrating the *r*-component of the force defined as

5 $${{\rm{\Psi }}}_{r}(r)={\int }_{0}^{r}{F}_{r}dr$$

For two interacting droplets, there are two opposite forces acting between them. Experimental observations suggest that there is a repulsion force acting between droplets, which keeps them at the distance apart from each other^1–5^. There is a viscous boundary layer in a gas at the droplet surface, which makes the velocity profile quite complex, so only a rough estimate is presented here. For the kinematic viscosity of ν = 2 × 10^−5^ m^2^ s^−1^, (air at 75 °C), distance between droplets *l* = 10^−4^ m and flow velocity of *U* = 0.1 ms^−1^, the value of the Reynolds number is estimated as Re = *Vl*/ν = 0.5. While large spheres in a fluid flow would tend to be attracted to each other, it is not necessary so for microspheres. Kim^16^, Folkersma^17^, and co-workers studied interaction between two identical spheres at Re = 50 and Re = 10 and found repelling force (which increased with decreasing Re), a weak repulsion force was also found even for Re = 10^−7^. It is therefore expected that such a repulsive force *F*(*d*) acts between two droplets in the ascending vapor-air flow.

An effective interaction potential dψ = *F*(*d*)d*d* can be introduced dependent on the distance between the droplets. This potential is imposed upon the potential created by the centripetal force directed towards the center of the hot spot. As a result, the droplets tend to accumulate towards the center of the hot spot with the tendency to maximize the distance between them minimizing the total effective energy given as

6 $${{\rm{\Psi }}}_{total}={{\rm{\Psi }}}_{r}({x}_{1},{y}_{1},\ldots ,\,{x}_{N},{y}_{N})+\sum _{\begin{array}{c}p=1,q=1\,\\ p\ne q\end{array}}^{N}\psi (\sqrt{{({x}_{p}-{x}_{q})}^{2}+{({y}_{p}-{y}_{q})}^{2}})$$

The minimum value of the total potential is supplied by the densest packing configuration, which is the hexagonal packing. The cluster is stable when the driving force towards the center prevails over the repulsion between the droplets.

We conclude that under the assumption of a quasi-steady temperature field, the repulsion force between the droplets acts in such a manner that they form the densest packing with the distance *L* between the droplets. Such packing is provided by the hexagonal pattern, which was observed experimentally. Potential application of the droplet cluster may involve various devices where aerosol droplet stabilization is needed, for example, for chemical analysis of droplet content. Understanding of the levitating microdroplet cluster behavior is important also for fundamental science since it forms an unusual relatively stable structure. Furthermore, microdroplets can catalyze various chemical reactions and thus serve natural micro-reactors^18^.
